# Gangrenous cholecystitis during extracorporeal membrane oxygenation operation: A case report and literature review

**DOI:** 10.3389/fmed.2023.1124863

**Published:** 2023-02-08

**Authors:** Peipei Wu, Shuai Wang, Qiao Gu, Ying Zhu, Wei Hu, Bingwei Liu

**Affiliations:** Department of Critical Care Medicine, Affiliated Hangzhou First People’s Hospital, Zhejiang University School of Medicine, Hangzhou, China

**Keywords:** extracorporeal membrane oxygenation, gangrenous cholecystitis, acute myocardial infarction, cardiopulmonary resuscitation, shock

## Abstract

A 50-year-old male presented to the emergency department of a hospital with an acute myocardial infarction who underwent cardiopulmonary resuscitation (CPR) followed by extracorporeal membrane oxygenation (ECMO). The patient developed persistent jaundice during the course of the disease, which was later found to be gangrenous cholecystitis. We believe this case report will alert clinicians to the possibility of this complication and encourage early detection and intervention to improve the prognosis. Traditionally, the gallbladder has received secondary attention in patients receiving ECMO support, as vital organs tend to be prioritized. However, this case report illustrates the importance of preserving gallbladder function in patients receiving ECMO support.

## Introduction

Extracorporeal membrane pulmonary oxygenation (ECMO) is a life support therapy for patients with cardiac or pulmonary failure, and its use is gradually expanding (1). The use of ECMO has gradually increased especially during the COVID-19 pandemic. However, with the increased use of ECMO, more and more refractory cases require longer-term ECMO support, during which various complications may occur that can seriously affect the prognosis. Gangrenous cholecystitis, also known as complicated cholecystitis is a manifestation when gangrene occurs in the gallbladder due to various causes, and has a very high mortality rate because clinicians are very prone to miss and misdiagnose the disease during the treatment process (2).

However, there are no reports of such complications during ECMO. We identified a case of this rare complication during ECMO operation that severely affected the patient’s prognosis, and we will report this rare complication to alert other physicians to the possibility of this rare complication and to analyze the cause.

## Case report

A 50-year-old male patient with chronic chest pain for 3 hours presented to the emergency department of a local hospital on September 4, 2021. The patient was diagnosed with an acute myocardial infarction after a thorough examination. The patient had persistent hypotension and weak awareness, indicating he was in cardiogenic shock. He was consequently referred to the Affiliated Hangzhou First People’s Hospital, followed by a sudden attack of ventricular tachycardia, ventricular fibrillation, and cardiopulmonary arrest with poor resuscitation. We performed extracorporeal membrane oxygenation (ECMO) and established extracorporeal life support after nearly 70 min of cardiopulmonary resuscitation. The patient was immediately given a coronary angiography, and ventricular fibrillation occurred during the procedure.

The patient had a history of hypertension and fatty liver disease. He had consumed alcohol for more than 30 years, averaging 250–500 ml of white wine per day.

**Figure 1:**
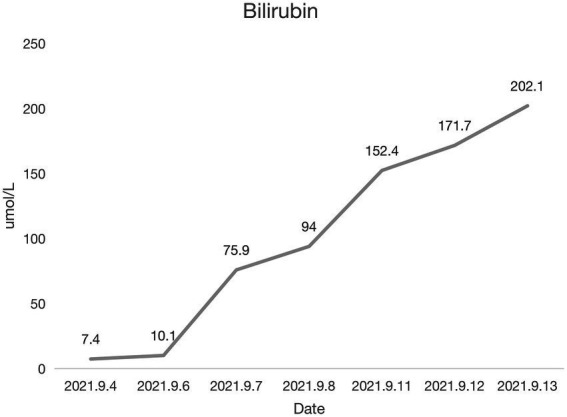
Bilirubin changes.

**Figure 2:**
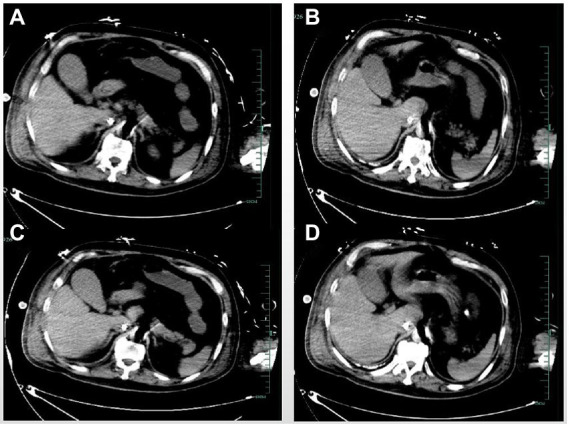
The abdominal computed tomography **(A–D)** levels showed no significant abnormality of the gallbladder.

**Figure 3:**
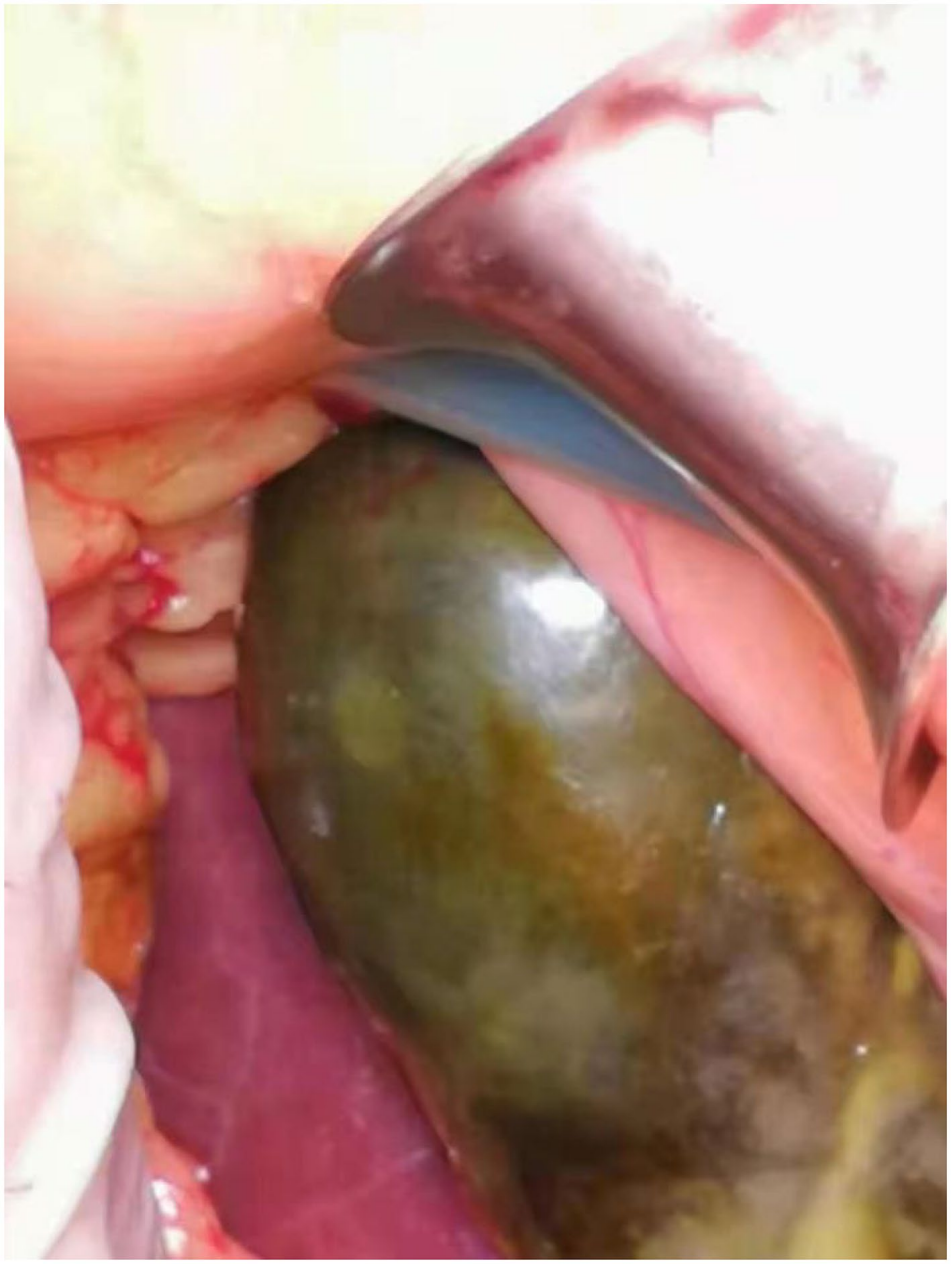
Gangrenous gallbladder visible on dissection.

Coronary angiography showed proximal complete occlusion of the anterior descending branch, complete occlusion of the right coronary artery opening, and 99% stenosis of the proximal circumflex coronary artery. The patient was transferred to the intensive care unit (ICU) after intra-aortic balloon pumping (IABP). The patient was admitted with a distended abdomen and a bladder pressure of 25 cmH_2_O to maintain circulation and treat myocardial infarction. The acid suppression with proton pump inhibitors was administered to the patient due to approximately 500 ml of coffee-like fluid draining from the gastric tube. The following biochemical index levels were present at admission: alanine aminotransferase (ALT) [143 U/l (0–40 U/l)], aspartate aminotransferase (AST) [917 U/l (0–40 U/l)], gamma-glutamyl transferase (GGT) [56 U/l (3–50 U/l)], alkaline phosphatase (ALP) [41 U/l (50–140 U/l)], total bilirubin (TBI) [7.4 μmol/l (3.4–17.1 μmol/l)], with the negative results of hepatitis A virus (HAV; IgG and IgM anti-HAV), hepatitis C virus (HCV; IgG and IgM anti-HCV), hepatitis E virus (HEV; IgG and IgM anti-HEV), hepatitis D virus (HDV; IgG and IgM anti-HDV), and hepatitis B surface antigen (HBsAg). Bedside ultrasound showed normal gallbladder size and wall and no dilatation of the intra- and extra-hepatic bile ducts. Elevated ALT and AST on admission was considered as liver injury due to ischemia and hypoxia. Liver perfusion pressure was maintained after admission. Some index values determined on September 7, 2021 were as follows: TBI-75.9 μmol/l, direct bilirubin (DBILI)-43.6 μmol/l (0–6.8 μmol/l), and indirect bilirubin (IBIL)-32.3 μmol/l (1.7–10.2 μmol/l). There was no significant change in the gallbladder on bedside ultrasound. We considered that the bilirubin might be elevated due to biliary stasis, so we opened enteral nutrition and added hepatoprotective medication. The following index levels were present on September 8, 2021: GGT-52 U/l, ALP-4 U/l, ALT-784 U/l, AST-2076 U/l, TBIL-94 μmol/l, DBILI-58.2 μmol/l, and IBIL-35.8 μmol/l, with normal ranges of prothrombin time (PT) and international normalized ratio (INR). After that, the TBI value fluctuated around 100 μmol/l, GGT and ALP levels remained normal, and daily ultrasound of the gallbladder revealed no significant abnormality, with the intra-abdominal pressure ranging from 20 to 30 mmH_2_O. The patient had no bowel movement. Therefore, he received continuous gastrointestinal (GI) decompression and intermittent enemas. The patient was changed to veno-arterial–venous (VAV)-ECMO on September 10, 2021(Right femoral artery, right femoral vein, right internal jugular vein), due to inadequate oxygenation, and his circulation and oxygenation functions gradually improved. The TBI level rose to 152.4 μmol/l on September 11, 2021 (Figure 1). We screened the portal vein and gallbladder ultrasounds and found an open portal vein with smooth flow and biliary stasis in the gallbladder. The patient did not wake up even after stopping sedative drugs. Abdominal CT (Figure 2) was performed with the assistance of ECMO and an intraaortic balloon pump (IABP). The multi-disciplinary teams (MDT) thought that the patient’s current liver injury and long-term hypoperfusion were caused by long-term alcohol consumption and alcoholic liver disease. The current hyperbilirubinemia should be considered intrahepatic cholestasis caused by long-term fasting and drug use. The gastroenterologist did not recommend endoscopic retrograde cholangiopancreatography because he thought that the patient had no signs of biliary obstruction or hemolytic jaundice. The patient showed high intra-abdominal pressure and poor intestinal motility. Therefore, we performed the cholecystectomy with ECMO support, and the pathologic finding suggested gallbladder gangrene (Figure 3). The patient’s cardiac function recovered poorly after surgery, and he died on the 16th day of ECMO treatment.

## Discussion

We described a patient on ECMO for cardiopulmonary arrest following acute myocardial infarction. The patient developed unexplained hyperbilirubinemia during the disease course and was determined to have gangrenous cholecystitis (GC) on Cesarean exploration, which has not been previously reported. We first reported this case with unexplained jaundice and explored the cause and diagnostic method of GC during ECMO assistance.

The most common cause of GC is gallbladder stone, followed by bacterial invasion, chemical inflammations from pancreatic juice reflux, and ischemia and gangrene of the gallbladder wall due to vascular changes from late arteritis (3). Risk factors of GC include advanced age, male gender, elevated white blood cell (WBC) count (> 17 × 10^9^/L), high C-reactive protein (CRP) level, alcohol abuse, cardiovascular disease, and diabetes mellitus. Cardiovascular disease is an independent risk factor for GC (4). The patient had no history of gallbladder stones, with no gallbladder stones on admission ultrasonography and subsequent abdominal CT. Therefore, we ruled out GC due to no evidence of gallbladder stones despite the male patient had a prior history of cardiovascular disease and alcohol use disorder. The patient’s bilirubin remained normal during the first 3 days of ECMO support, and the abnormal liver function might be attributed to ischemic liver injury caused by hypoperfusion. Additionally, the following issues remained unclear, including whether the gallbladder injury was due to hypoperfusion, the biliary stasis was caused by prolonged fasting, and the gallbladder infection was attributed to the transference of intestinal flora. We will discuss them below.

There is prolonged hypoperfusion in patients undergoing extracorporeal CRP (ECPR) at an early stage, leading to organ ischemia and lower blood flow in the digestive system. Considering the gallbladder artery is mainly supplied from the right hepatic artery, the above conditions can cause gallbladder ischemia and ischemia–reperfusion injury (IRI). It is also possible that this has led to embolism of the gallbladder artery. The incidence of acute GI injury is high in critically ill patients, ranging from 40 to 90% (5). It is caused by changes in intestinal ischemia and hypoxia due to various etiological strikes. Moreover, intestinal IRI can cause structural and functional alterations in the intestinal mucosa, including damages to mucosal epithelial cells, tight junctions, and mucus barrier, mucosal ischemia, increased permeability, intestinal wall edema, impaired GI motility, altered ecological environment in the intestinal lumen, and intestinal flora displacement (6). The patient developed gastrointestinal bleeding after prolonged hypoperfusion. We performed the gastroscopy after a rigorous discussion and found a gastric ulcer, indicating the patient had an impaired mucosal barrier. Therefore, we should prevent prolonged hypoperfusion in those with high-risk factors for GC and alert the complication in the event of bilirubin elevation.

Enteral nutrition may be one of the reasons for this. Water fasting can cause decreased cholecystokinin secretion, defective gallbladder emptying, and biliary stasis. Bile sludge can stimulate the gallbladder epithelium to secrete inflammatory mediators like prostaglandin and interleukin, resulting in increased edema and congestion of the gallbladder mucosa, increased intra-biliary pressure, decreased blood supply to the gallbladder wall, and ischemic gangrene and perforation (7). Guidelines recommend enteral nutrition (EN) should be started within 24–48 h of admission to the intensive care unit (ICU) if a patient is unable to eat (8). However, we decided to extend the eating time due to the high incidence of the patient’s gastrointestinal intolerance and hemodynamic instability. We started experimental EN on the fifth day after admission. However, it was suspended again because of the patient’s high intra-abdominal pressure and poor intestinal motility. We previously prioritized the gastrointestinal tract after the heart, lung, brain, and kidney due to the superior tolerance of the gastrointestinal tract and rare reports of gastrointestinal perforation during ECMO (9). We suspected that prolonged fasting may induce GC. The European Society of Intensive Care Medicine (ESICM) 2017 clinical practice guidelines recommended delayed EN if the shock is not controlled with inadequate hemodynamic and tissue perfusion. Instead, EN should not be delayed with the inotropic drug use, prone ventilation, and hypothermia, and it should be started at a low dose once the shock is managed with fluids and vasoactive medications (10). Therefore, we should be more active with EN during ECMO, even in small doses.

Analgesic and sedative drugs may be another cause. Sedation and analgesia are crucial in managing patients on ECMO. Contrary to patients on mechanical ventilation, the optimal sedation and analgesia regimen is not established for patients on ECMO. Extracorporeal Life Support Organization (ELSO) recommends gentle sedation of patients to reduce oxygen consumption and optimize ventilation during intubation and for 12–24 h after intubation (11). Opioid analgesics act with the opioid receptors in the central nervous system. However, the matching antibodies are distributed in the enteric nerves, which may increase the tension of pyloric and choledochal sphincters and decrease the intestinal juice, bile, and pancreatic secretions (12). The analgesic and sedative drugs may affect the gallbladder motility and Oddi sphincter, leading to reduced gallbladder contraction and bile outflow activities. Increased intra-biliary pressure, gallbladder distention, increased gallbladder tension, reduced blood flow, hemorrhage, and gallbladder necrosis and abscess are the results of these circumstances (13). We generally administer analgesic and sedative drugs during ECMO to reduce organ oxygen consumption and avoid dislodging the ducts. We frequently overlook the effect of sedative drugs on the gallbladder. Therefore, we should reduce drug use and avoid adverse effects immediately in patients with stable hemodynamics.

Early detection and surgery are essential for treating GC. Patients undergoing ECMO after ECPR mostly have a longer ischemic–hypoxic time and encephalopathy. Patients may develop pain insensitivity and frequently require sedative and analgesic drugs, making the early identification of cholecystitis difficult. Therefore, systematic monitoring and multiple imaging methods are necessary for diagnosing acute cholecystitis.

Imaging techniques are vital in diagnosing acute cholecystitis. They can provide information on gallbladder size, gallbladder wall thickness, gallbladder lumen, gallbladder duct, and gallbladder stone. Abnormalities within the gallbladder lumen (mucosal detachment, bleeding, and gas), gallbladder wall abnormalities (streaking, asymmetric wall thickening, gas, and loss of reflection of ultrasound and contrast enhancement), and peri-gallbladder changes (fatty echogenicity, peri-gallbladder fluid, and abscess) can help diagnose GC (14). Ultrasonography is the most popular imaging modality for acute cholecystitis. It is convenient, economical, noninvasive, and radiation-free. Besides, it can quantify the long and short gallbladder axes, evaluate the stratification of gallbladder wall thickness, and detect stone impaction, echogenic debris, and peripheral fluid accumulation (15). The available findings show that the ultrasound features of GC are intraluminal membrane conditions, such as mucosal detachment, ulcer, and fibrous exudate. Focal wall extrusion, ulceration, and rupture may be visible on ultrasound. The above signs are mostly associated with an increased risk of gallbladder perforation. Nevertheless, most GC patients exhibit a negative Murphy’s sign, which may be related to denervation and gallbladder wall gangrene (16, 17). Moreover, the imaging of ultrasound may be affected by the complexity of the abdomen. Ultrasonography is a specific test for GC. It typically manifests as discontinuous and irregular enhancements of the gallbladder wall. A previous study showed that the accuracy rate of ultrasonography in detecting GC could reach 83% (18). Therefore, it is crucial to monitor the gallbladder with ultrasound during ECMO. However, we did not detect any GC signs with daily bedside ultrasound, indicating the need for additional adjuvant examinations.

CT may be another option. However, studies (14) have shown that CT plain scan is less accurate than ultrasonography in diagnosing acute cholecystitis. CT can assess the overall disease severity and guide the treatment in patients with complicated cholecystitis, such as gangrene, hemorrhage, and perforation. Maehira et al. (19) found that the liver showed more pronounced transient focal enhancement in GC patients than in non-GC patients on arterial phase-enhanced CT. However, CT is constrained in patients on ECMO due to the high transport risk. Moreover, enhanced CT may be challenging for patients receiving ECMO because of the altered blood flow. The abdominal CT was performed in our case. However, no signs of GC were found (Figure 2). Multiple examinations may be necessary to determine GC.

Hepatobiliary surgeons are exploring diagnostic markers for GC. CA199 was previously used as a marker in diagnosing pancreatic cancer. However, some investigators have recently discovered that CA199 could be significantly elevated in patients with cholecystolithiasis with cholecystitis and GC and returned to the normal range in postoperative follow-up (20). However, these are only case reports, and more studies are needed to verify them. It is inherently difficult to diagnose GC, especially in patients on ECMO. Therefore, such patients can easily develop gallbladder perforation with a serious outcome. Cholecystitis was not detected by ultrasound and CT in this patient. Fortunately, GC was discovered because of a cholecystectomy.

In conclusion, the patient of unexplained jaundice on ECMO was finally diagnosed as GC, which is a very rare and fatal complication. We should alert this disease and detect and intervene early to improve the patient’s prognosis and reduce the GC possibility. Our prior attention to the gastrointestinal tract and gallbladder of the patients on ECMO was minimal. With the maturation of ECMO technology, we should focus on protecting gallbladder function and preventing GC occurrence in patients with longer ECMO support.

## Data availability statement

The original contributions presented in the study are included in the article/supplementary material, further inquiries can be directed to the corresponding authors.

## Ethics statement

Written informed consent was obtained from the individual(s) for the publication of any potentially identifiable images or data included in this article.

## Author contributions

All authors listed have made a substantial, direct, and intellectual contribution to the work and approved it for publication.

## Funding

This study was funded by the Construction Fund of Medical Key Disciplines of Hangzhou (Grant: OO20200485).

## Conflict of interest

The authors declare that the research was conducted in the absence of any commercial or financial relationships that could be construed as a potential conflict of interest.

## Publisher’s note

All claims expressed in this article are solely those of the authors and do not necessarily represent those of their affiliated organizations, or those of the publisher, the editors and the reviewers. Any product that may be evaluated in this article, or claim that may be made by its manufacturer, is not guaranteed or endorsed by the publisher.
